# Effect of Community Health Workers on 30-Day Hospital Readmissions in an Accountable Care Organization Population: A Randomized Clinical Trial

**DOI:** 10.1001/jamanetworkopen.2021.10936

**Published:** 2021-05-20

**Authors:** Jocelyn Carter, Susan Hassan, Anne Walton, Liyang Yu, Karen Donelan, Anne N. Thorndike

**Affiliations:** 1 Department of Medicine, Massachusetts General Hospital, Boston; 2 Division of General Internal Medicine, Massachusetts General Hospital, Boston; 3 Mongan Institute, Massachusetts General Hospital, Boston; 4 Brandeis University, Waltham, Massachusetts; 5 Division of General Internal Medicine, Massachusetts General Hospital, Harvard Medical School, Boston

## Abstract

**Importance:**

Value-based care within accountable care organizations (ACOs) has magnified the importance of reducing preventable hospital readmissions. Community health worker (CHW) interventions may address patients’ unmet psychosocial and clinical care needs but have been underused in inpatient and postdischarge care.

**Objective:**

To determine if pairing hospitalized patients with ACO insurance with CHWs would reduce 30-day readmission rates.

**Design, Setting, and Participants:**

This randomized clinical trial was conducted in 6 general medicine hospital units within 1 academic medical center in Boston, Massachusetts. Participants included adults hospitalized from April 1, 2017, through March 31, 2019, who had ACO insurance and were at risk for 30-day readmission based on a hospital readmission algorithm. The main inclusion criterion was frequency of prior nonelective hospitalizations (≥2 in the past 3 months or ≥3 in the 12 months prior to enrollment). Data were analyzed from February 1, 2018, through March 3, 2021.

**Intervention:**

CHWs met with intervention participants prior to discharge and maintained contact for 30 days postdischarge to assist participants with clinical access and social resources via telephone calls, text messages, and field visits. CHWs additionally provided psychosocial support and health coaching, using motivational interviewing, goal-setting, and other behavioral strategies. The control group received usual care, which included routine care from primary care clinics and any outpatient referrals made by hospital case management or social work at the time of discharge.

**Main Outcomes and Measures:**

The primary outcome was 30-day hospital readmissions. Secondary outcomes included 30-day missed primary care physician or specialty appointments.

**Results:**

A total of 573 participants were enrolled, and 550 participants (mean [SD] age, 70.1 [15.7] years; 266 [48.4%] women) were included in analysis, with 277 participants randomized to the intervention group and 273 participants randomized to the control group. At baseline, participants had a mean (SD) of 3 (0.8) hospitalizations in the prior 12 months. There were 432 participants (78.5%) discharged home and 127 participants (23.1%) discharged to a short rehabilitation stay prior to returning home. Compared with participants in the control group, participants in the intervention group were less likely to be readmitted within 30 days (odds ratio [OR], 0.44; 95% CI, 0.28-0.90) and to miss clinic appointments within 30 days (OR, 0.56; 95% CI, 0.38-0.81). A post hoc subgroup analysis showed that compared with control participants, intervention participants discharged to rehabilitation had a reduction in readmissions (OR, 0.09; 95% CI, 0.03-0.31), but there was no significant reduction for those discharged home (OR, 0.68; 95% CI, 0.41-1.12).

**Conclusions and Relevance:**

This randomized clinical trial found that pairing ACO-insured inpatient adults with CHWs reduced readmissions and missed outpatient visits 30 days postdischarge. The effect was significant for those discharged to short-term rehabilitation but not for those discharged home.

**Trial Registration:**

ClinicalTrials.gov Identifier: NCT03085264

## Key Points

**Question:**

Does community health worker care reduce 30-day hospital readmissions in inpatient adults participating in accountable care organization plans?

**Findings:**

In this randomized clinical trial including 550 adults, intervention patients were significantly less likely to experience 30-day hospital readmissions than control participants. In post hoc subgroup analysis, the effect remained significant for participants discharged to short-term rehabilitation but not for those discharged home.

**Meaning:**

In this study, community health workercare improved postdischarge outcomes in clinically complex patients insured by accountable care organizations, particularly for those discharged to short-term rehabilitation.

## Teaser

This randomized clinical trial examines the effect of community health worker care on hospital readmissions within 30 days for adults enrolled in an accountable care organization.

## Introduction

Hospitalizations account for one-third of US health care costs.^1,2^ Readmissions in the first 30 days after hospital discharge are common, and approximately 27% of adult 30-day readmissions are estimated to be preventable.^3^ Increasing movement toward value-based care within accountable care organizations (ACOs) has magnified the importance of reducing preventable readmissions.^4^ Factors associated with hospital readmissions include medical complexity,^5^ clinical comorbidities,^6,7^ and social determinants of health.^8,9,10^ Many health care organizations have increasing interest in developing interventions within the ACO framework that address clinical care gaps and unmet social needs. Integration of community health workers (CHWs) is one of few interventions that have generated promising outcomes in terms of reducing hospitalizations and health care costs.^11,12,13^

CHWs are trained to have basic knowledge of clinical conditions and to provide health coaching using motivational interviewing and psychosocial support.^14,15,16^ CHWs also have expertise in social determinants of health and can assist patients with economic, health care access–associated, social, educational, and environmental resources to help close gaps in patient health care.^17,18^ Specifically, CHWs can augment patient engagement by strengthening patient connections to primary care and identifying relevant community-, state-, or federal-based patient resources to meet unmet patient needs (eg, food, housing, transportation).^19,20^

While most CHW studies have focused on specific disease-based cohorts,^21,22,23,24,25,26^ some randomized clinical trials have examined the effectiveness of using CHWs to improve postdischarge outcomes in adult inpatient and outpatient general medicine populations. In a study of 222 Medicaid inpatients randomized to a CHW intervention, Kangovi et al^27^ found that having at least one 30-day readmission did not differ between the intervention and control groups; however, there was a significant reduction in the number of intervention participants with 2 or more 30-day readmissions. In a randomized clinical trial of 1009 inpatients older than 60 years, Balaban et al^28^ found that CHW care reduced 30-day readmission rates in intervention participants compared with control participants. Another randomized clinical trial by Kangovi et al^29^ of 592 primary care outpatients tested a 6-month CHW intervention and demonstrated a reduced likelihood of readmission. Two other randomized clinical trials examining 6-month interventions with CHWs demonstrated no significant difference in hospitalizations.^30,31^

To determine the effect of CHW care delivery on 30-day readmissions within an ACO population, we conducted a randomized clinical trial to test a 30-day CHW intervention for patients admitted to the internal medicine service in an academic medical center in Boston, Massachusetts. The hypothesis was that CHW care delivery initiated in the hospital and extending for 30 days after discharge would reduce 30-day readmissions compared with usual care.

## Methods

This randomized clinical trial was approved by the of Partners Human Research Committee (Trial Protocol in Supplement 1; eAppendix in Supplement 2). All enrolled patients provided written informed consent for study enrollment. This study is reported following the Consolidated Standards of Reporting Trials (CONSORT) reporting guideline for randomized clinical trials.

### Trial Design

The Community Care Transitions study was a randomized clinical trial was designed to improve health care outcomes for patients at high risk for readmission by pairing CHWs with inpatients for 30 days after discharge.^32^ CHW care delivery was implemented in partnership with clinical teams that were unaware of outcomes until trial completion. The CHWs addressed and integrated patient-identified needs (eg, food, housing, transportation) into the clinical care plans and used motivational interviewing and psychosocial support strategies to improve adherence to clinical care.

### Setting and Participants

The study was conducted at Massachusetts General Hospital, a 999-bed teaching hospital in Boston, Massachusetts. Six internal medicine hospital units (or clinical wards) were used for trial recruitment. Each unit had similar percentages of 30-day readmissions with no differences in the diagnoses or ages of hospitalized patients.

**Figure 1.**
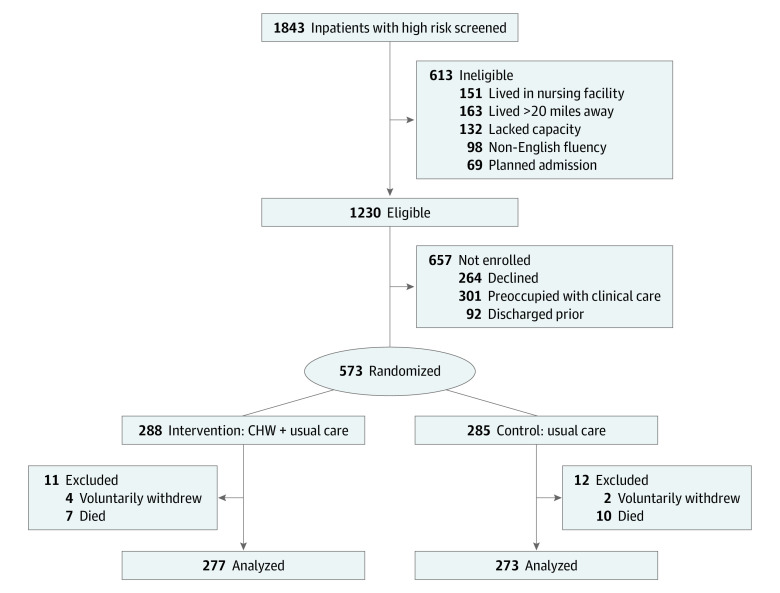
Participant Recruitment Flowchart CHW indicates community health worker.

Eligibility criteria were developed based on findings from 2 prior studies by Carter et al.^33,34^ Briefly, patients aged 18 years or older who were admitted to 1 of the study units were potentially eligible if they met the high-risk hospital criteria for 30-day readmission, with threshold of 16% or greater readmission risk. This was determined by a Massachusetts General Hospital–based 12-factor risk algorithm that included prior hospitalizations, fall risk, wound care needs, and frailty. After being identified by this algorithm, patients were evaluated by research staff for additional study inclusion criteria: prior history of 2 or more nonelective hospitalizations in the 3 months prior to enrollment or 3 or more nonelective hospitalizations in the 12 months prior to enrollment; participation in a hospital-based ACO benefit (Medicare, Medicaid, or private insurance); living within a 20-mile radius of the main hospital; having access to a working telephone; being fluent in English; having 1 or more unmet care-related needs identified during inpatient multidisciplinary rounds (eg, difficulties with medication management, appointment scheduling, access to transportation, or social support); and having a primary care physician (PCP). Patients were ineligible if they were experiencing homelessness, unable to provide consent owing to cognitive impairment, or had an invoked health care proxy or prisoner status (Figure 1). Patients with preexisting outpatient program support (ie, integrated care management services with telephonic nursing, social work, and clinical resource care coordination), home nursing, or other supportive programming (eg, physical therapy) were eligible for participation in the trial. Patients living in nursing homes or discharged to long-term care were excluded prior to randomization.

### Enrollment and Randomization

Patients were identified with a hospital-based database that generated a daily report of inpatients at high risk for readmission. Eligible patients were discussed at multidisciplinary rounds daily on each inpatient unit with CHW staff, case managers, unit nurses, bedside nurses, and physicians who reviewed the potential barriers to discharge. After confirming eligibility, research staff approached patients for enrollment. After providing consent and agreeing to enrollment, patients were randomized by study staff via block randomization using the team statistician’s preloaded Excel spreadsheet version 16.0 (Microsoft) block with 6 participants per block to either the intervention or the control group. To allow enough time for enrollment procedures, most participants had to be enrolled prior to finalization of their discharge plans; therefore, it was not feasible to preidentify which participants would be discharged to home or to a short-term rehabilitation facility.

### Intervention Care

CHWs met with intervention participants and inpatient multidisciplinary teams prior to discharge to establish goals identified by patients and clinical teams. After discharge, CHWs delivered care using multiple communication strategies, including telephone calls, text messages, home visits, rehabilitation facility visits, and field visits (eg, accompanying patient for medical or social service encounters). CHWs provided health coaching, as well as assistance with obtaining any needed clinical access or social resources. CHWs were trained in CHW core competencies,^19^ consisting of motivational interviewing, goal-setting, behavior change, and psychosocial support.

Three CHWs delivered the study intervention, and all had experience working with or living in communities similar to those of participants. All CHWs received 1 month of protocol training led by the study CHW supervisor. CHW core competencies were emphasized during training and applied to case-based scenarios. CHWs documented all encounters in the electronic medical record (EMR) (eg, enrollment notes, progress notes for in-person visits and telephone calls); all patient and care team interactions were documented by CHWs in a REDCap database (Vanderbilt University). Each patient’s clinical team members were copied on all CHW EMR notes and contacted directly by CHWs during the intervention when needed. CHW staff also communicated directly with any practitioners of each patient’s outpatient primary care based–support, such as care management, nursing, or physical therapy, to streamline care delivery.

### Usual Care

Usual care was defined as routine postdischarge care from the hospital discharge team. Any outpatient referrals made by hospital case management (eg, visiting nursing association, physical therapy, occupational therapy) or social work (eg, meal preparation, transportation, elder care services) at the time of discharge were also considered part of usual care.

### Measures

Study participants completed an enrollment questionnaire adapted from a previous survey instrument.^26^ This survey instrument was derived from some standard established measures of patient experience for benchmarking as well as validated questions generated by prestudy qualitative interviews with patients and physicians. Questionnaire domains included health-related social needs (eg, food, housing, transportation), perceptions of their physical and mental health, confidence in their ability to care for themselves after discharge, satisfaction with inpatient care, perceived likelihood of readmission, understanding of the care plan, and ability to independently perform activities of daily living. Basic demographic characteristics, insurance status, primary diagnosis associated with admission, and major medical and psychiatric comorbidities were collected by EMR review. All participants were asked to complete a 30-day postdischarge questionnaire that included questions to assess perceived likelihood of 30-day readmission and confidence in caring for oneself outside the hospital.

### Outcomes

The primary outcome was 30-day hospital readmission during the study period. Prespecified secondary outcomes were 30-day postdischarge missed outpatient physician appointments and emergency department (ED) visits. Outcomes were determined by the number of participants with 1 or more hospital admissions, ED visits (including ED observation stays but excluding ED visits resulting in hospitalization), or missed appointments during the 30 days after discharge from the index hospitalization. Automated email notifications of Partners Healthcare admissions and ED visits were generated by a hospital readmissions database and sent to study staff within 1 hour of occurrence. Hospital admissions were captured at Partners Healthcare–affiliated hospitals in Massachusetts (3 academic hospitals; 6 community hospitals) and New Hampshire (1 community hospital). Missed appointments were captured similarly by a hospital database and tabulated quarterly. Since the EMR was unable to capture encounters outside Partners Healthcare, participants were asked about clinical encounters during a 30-day postdischarge questionnaire. Owing to the nature of the intervention, patients and CHW staff were unable to be blinded to the study treatment arms. Study staff collecting EMR and patient-reported outcomes and performing data analysis were blinded. Clinical outcomes were adjudicated independently of the trial.

### Power Calculation

For the power calculation, we assumed a readmission rate of 18% for usual care and a readmission rate of 13% with use of CHW care delivery, requiring a sample size of 1200 adults (600 intervention and 600 control) with more than 90% power to reject the null hypothesis that the readmission rate was greater than 13% using 1-sided binomial testing with 5% type I error. However, we were unable to enroll 1200 patients within the study funding timeline; therefore, recruitment was stopped prior to achieving the planned sample size.

### Statistical Analysis

Demographic characteristics and baseline survey item responses were summarized between intervention and control groups. Bivariate analyses, using Pearson χ^2^ tests for categorical variables and *t* test for continuous variables, were performed to assess differences between binary clinical outcomes (30-day hospital readmission, missed appointments, ED visits) and study arms, as well as demographic characteristics and survey item responses. For every clinical outcome, a logistic regression model was applied to obtain unadjusted and adjusted odds ratios (ORs) to assess the intervention effect. Covariates adjusted for in the model included age, race/ethnicity, sex, number of hospitalizations, insurance, living alone status, and discharge disposition. Race/ethnicity was self-reported and collected as a potential confounder of readmissions, missed appointments, and ED visits. For 30-day patient experience outcomes, patient responses at admission and 30 days after discharge were compared, and a difference-in-differences analysis was performed. *P* values were 2-sided, and *P* < .05 was considered statistically significant. A separate univariate analysis was performed to identify types of CHW-patient contact along with categories of resources or care delivery administered. All analyses were performed using SAS statistical software version 9.4 (SAS Institute). Data were analyzed from February 1, 2018, through March 3, 2021.

## Results

Patients were enrolled April 1, 2017, through March 31, 2019. A total of 1843 patients at high risk for readmission were screened, 1230 patients were deemed eligible, and 573 patients were randomized to the intervention (288 patients) or control (285 patients) groups (Figure 1). Six participants (1.0%), including 4 from the intervention group and 2 from the control group, withdrew from the study, and 17 participants (3.1%), including 7 from the intervention group and 10 from the control group, died prior to completing the study and were excluded. The remaining 550 participants were included in the final analyses, with 277 participants in the intervention group and 273 participants in the control group.

The mean age (SD) of participants was 70.1 (15.7) years, and 266 (48.4%) were women (Table 1). The most common insurance was Medicare (388 participants [70.5%]). All trial participants had a mean (SD) of 3 (0.8) hospitalizations in the 12 months prior to index hospitalization (Table 1). Overall, 67 participants (24.5%) in the control group and 60 participants (21.7%) in the intervention group were discharged to rehabilitation. The mean (SD) length of participant rehabilitation stay was 3.9 (1.1) days. Identified covariates were balanced in intervention and control groups.

**Table 1.** Patient Characteristics

Patient characteristics	No. (%)	
	Control (n = 273)	Intervention (n = 277)
Sex		
Women	119 (43.6)	147 (53.1)
Men	154 (56.4)	130 (46.9)
Age, mean (SD), y	69.7 (16.1)	70.4 (15.3)
Race/ethnicity		
Hispanic/Latino	7 (2.6)	9 (3.2)
White	253 (92.7)	241 (87.0)
Black	10 (3.7)	24 (8.7)
Asian	3 (1.1)	2 (0.7)
Other^a^	0 (0.0)	1 (0.4)
≤High school education	123 (45.1)	140 (50.5)
Primary insurance		
Medicare	191 (70.0)	197 (71.1)
Medicaid or MassHealth	34 (12.5)	30 (10.8)
Commercial or private	48 (17.6)	50 (18.1)
Preexisting services		
Comprehensive case management	113 (41.4)	119 (43.0)
Clinical nursing or home services	76 (27.8)	70 (25.3)
Social determinants		
Lives alone	90 (33.0)	94 (33.9)
Housing quality problems (eg, leaks, poor heat/cooling, insects)	27 (10.0)	21 (7.6)
Had trouble paying in the last 12 mo		
Medical bills	24 (8.8)	29 (10.5)
Prescription drugs	36 (13.2)	40 (14.4)
Medical equipment or supplies	10 (3.7)	22 (7.9)
Health care services at home	10 (3.7)	10 (3.6)
Food	39 (14.3)	46 (16.6)
Clothing	33 (12.1)	38 (13.7)
Rent, mortgage, or housing costs	27 (9.9)	36 (13.0)
Inability performing ≥2 ADL independently	121 (44.3)	137 (49.5)
Healthcare utilization		
No. of hospitalizations within 12 mo, mean (SD)	3.0 (0.7)	3.1 (0.9)
Primary reason hospitalization		
Infectious disease	78 (28.6)	63 (22.7)
Gastroenterology condition	47 (17.2)	60 (21.7)
Cardiac	58 (21.2)	62 (22.4)
Respiratory condition	24 (8.8)	24 (8.7)
Fall or trauma	21 (7.7)	11 (4.0)
Other	45 (16.5)	57 (20.6)
Disposition at discharge		
Home	206 (75.5)	217 (78.3)
Rehabilitation	67 (24.5)	60 (21.7)

Abbreviation: ADL, activities of daily living.

^a^ Includes American Indian/Alaska Native, Asian, Native Hawaiian or other Pacific Islander, unknown or not reported, and other.

**Figure 2.**
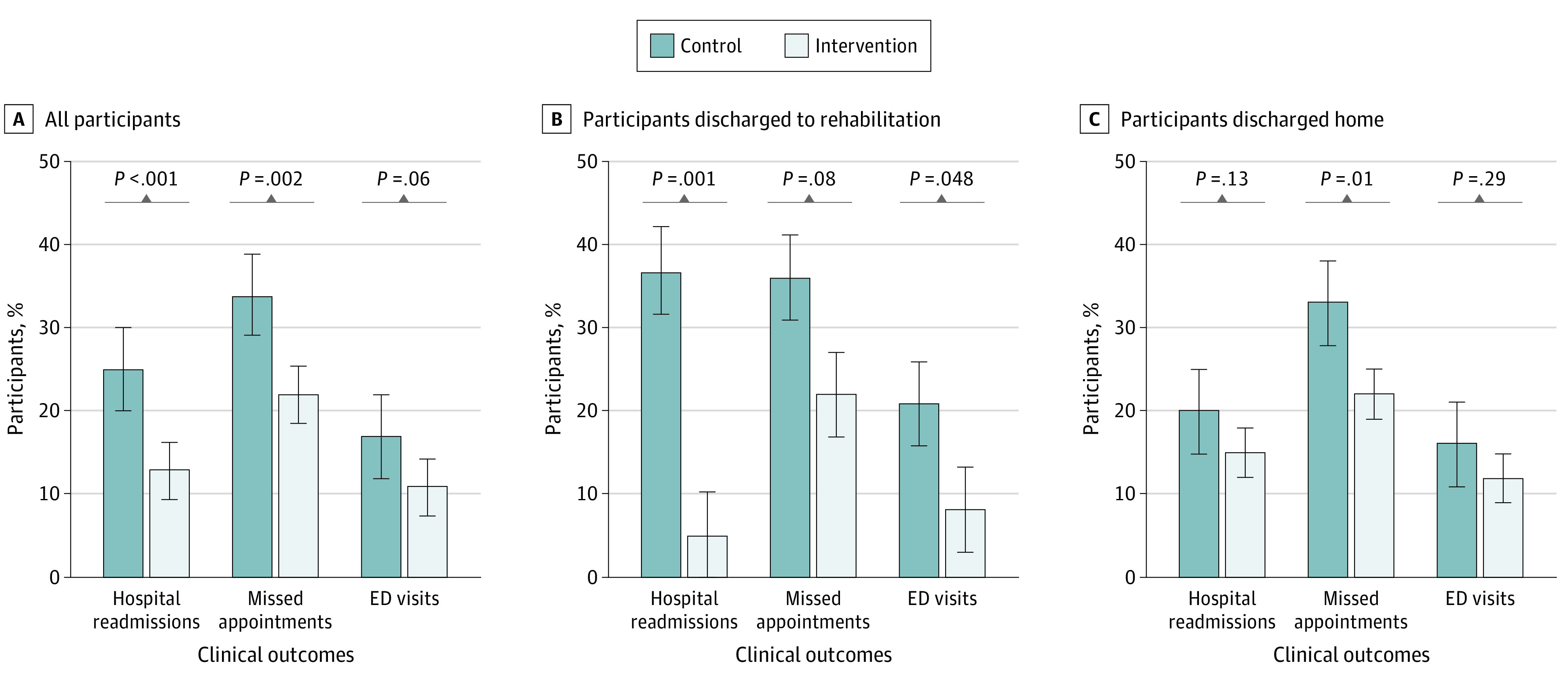
Postdischarge Outcomes at 30 Days for Participants Paired With Community Health Workers vs Usual Care Adjusted for age, race/ethnicity, sex, number of hospitalizations, insurance, and living alone; discharge disposition was also applied for panel A only. ED indicates emergency department; error bars, 95% CI.

Overall, compared with the control group, fewer participants in the intervention group were readmitted in the 30 days after hospital discharge (67 participants [24.5%] vs 35 participants [12.6%]; OR, 0.44; 95% CI, 0.28-0.90; *P* < .001) (Figure 2A). In a post hoc subgroup analysis of this cohort (eTable 2 in Supplement 2), we found that intervention participants discharged to rehabilitation demonstrated a reduction of 32.3 percentage points in readmissions compared with control participants (3 participants [5.0%] vs 25 participants [37.3%]; OR, 0.09; 95% CI, 0.03-0.31; *P* < .001) (Figure 2B), but the difference observed in intervention vs control participants discharged home was not statistically significant (32 participants [14.7%] vs 42 participants [20.4%]; OR, 0.68; 95% CI, 0.41-1.12; *P* = .13) (Figure 2C). A total of 4 readmissions occurred outside the hospital system and were identified by the patient questionnaire.

Fewer intervention than control participants had missed appointments (61 participants [22.0%] vs 92 participants [33.7%]; OR, 0.56; 95% CI, 0.38-0.81; *P* = .002), but the difference in ED visits was not statistically significant (31 participants [11.2%] vs 46 participants [16.8%]; OR, 0.62; 95% CI, 0.38-1.02; *P* = .06) (Figure 2A). Similar effects were observed in the subgroup analysis among intervention vs control participants discharged to rehabilitation (missed appointments: 13 participants [21.7%] vs 24 participants [35.8%]; OR, 0.5; 95% CI, 0.22-1.09; *P* = .08; ED visits: 5 participants [8.3%] vs 14 participants [20.9%]; OR = 0.34; 95% CI, 0.12-1.02; *P* = .05) (Figure 2B) or to home (missed appointments: 48 participants [22.1%] vs 68 participants [33.0%]; OR,  = 0.58; 95% CI, 0.37-0.89; *P* = .01; ED visits: 26 participants [12.0%] vs 32 participants [15.5%]; OR, 0.74; 95% CI, 0.42-1.29; *P* = .30) (Figure 2C). In multivariate analyses of all participants controlling for demographic and clinical covariates (ie, age, race/ethnicity, sex, number of hospitalizations, insurance, living alone status, and discharge to home vs rehabilitation), the adjusted ORs for clinical outcomes in intervention vs control participants were similar to the unadjusted ORs (30-day hospital readmissions: adjusted OR, 0.45; 95% CI, 0.29-0.72; missed appointments: adjusted OR, 0.56; 95% CI, 0.38-0.82; ED visits: adjusted OR, 0.62; 95% CI, 0.38-1.02) (eTable 1 in the Supplement). A similar analysis was performed for participants discharged home and to short-stay rehabilitation (eTable 3 and eTable 4 in the Supplement).

More than 80% of all participants indicated they were very or somewhat confident in caring for themselves at the time of enrollment, including 234 participants (84.5%) in the intervention group and 240 participants (87.9%) in the control group, and this did not change in their poststudy questionnaire (Table 2). Intervention participants demonstrated a decrease in their perceived likelihood of 30-day readmission in the poststudy questionnaire compared with the enrollment questionnaire (24 participants [12.5%] vs 53 participants [19.1%]; *P* = .04); this difference was not statistically different from the control group.

**Table 2.** Patient-Reported Experience Outcomes at 30 Days

Outcome	No.	No. (%)		Difference, percentage points	*P* value	Difference in differences, %		*P* value
		Admission	Poststudy					
Confident in caring for self (very/somewhat)								
Intervention	192	234 (84.5)	159 (82.8)	1.7	.43	1.0		.84
Control	196	240 (87.9)	167 (85.2)	2.7	.32			
Likelihood of 30-d readmission (very/somewhat)								
Intervention	192	53 (19.1)	24 (12.5)	6.6	.04	2.9		.38
Control	196	56 (20.5)	33 (16.8)	3.7	.26			

Intervention participants had a mean (SD) of 3.2 (2.4) contacts with CHWs after hospital discharge during the 30-day study period. Of 277 intervention participants, 247 (88.9%) communicated with CHW staff during at least 1 phone call. In addition, 198 participants (71.4%) in the intervention group received at least 1 home, rehabilitation, or field visit during the study interval (Table 3). CHWs completed different types of interactions focused on medical, social, or basic needs and coaching or education. CHWs most commonly provided participants with counseling to reinforce adherence to their clinical care plans (239 participants [86.3%]) and with psychosocial support (229 participants [82.7%]). Other CHW-patient activities ranged widely across supportive clinical and social domains (Table 3), including making and confirming clinical appointments (128 participants [46.2%]), assistance with securing basic needs like food or housing (108 participants [39.1%]), helping with access to medications (97 participants [35.0%]), creating plans for reliable transportation (91 participants [32.9%]), initiating elder care services (80 participants [28.9%]), engaging case management support (76 participants [27.4%]), and assisting with completing insurance forms or obtaining benefits (75 participants [27.0%]).

**Table 3.** Types of CHW-Patient Contacts and Activities for Intervention Participants

Activity	No. (%)
Contacts	
Phone visit with patient or caregiver	247 (89.2)
Direct patient contact	198 (71.4)
Home visit	139 (50.1)
Rehabilitation facility visit	31 (11.1)
Field visit (clinical or social support appointment)	28 (10.1)
Activities	
Medical needs	
Reinforcement of general adherence to care plans and medication	239 (86.3)
Making or confirming clinical appointments	128 (46.2)
Direct interaction with clinical care team member	111 (40.1)
Arranging for access to medications (delivery or transportation)	97 (35.0)
Engaging case management support	76 (27.4)
Completion of forms associated with unmet insurance needs	75 (27.0)
Social or basic needs	
Securing basic needs (eg, housing, food, electricity)	108 (39.1)
Creating a reliable transportation plan	91 (32.9)
Referral to a social service agency or program	85 (30.7)
Referral to elder services	80 (28.9)
Coaching or teaching	
Providing psychosocial support	229 (82.7)
Organization and reconciliation (eg, calendar events, mail, bills)	87 (31.4)
Nutrition and general health	39 (14.1)

Abbreviation: CHW, community health worker.

## Discussion

In this randomized clinical trial at 1 academic medical center, a CHW intervention reduced 30-day hospital readmissions in adult general medicine inpatients by nearly 50%. However, subgroup analyses revealed that most of the effect occurred for participants initially discharged to short-term rehabilitation. Intervention participants also were less likely to miss clinic appointments, but no significant reductions in ED visits were noted. These results indicate that CHW interventions may help reduce hospital readmissions and improve preventive care among some clinically complex patients within an ACO.

A subgroup analysis of participants who were discharged directly home compared with those discharged to short-term rehabilitation before going home demonstrated that the CHW intervention effect on 30-day readmissions was large for participants who went to rehabilitation. Prior studies have shown that deconditioned patients with complex comorbidities who are discharged to rehabilitation facilities have elevated rates of readmission, ranging from 28% to 75%.^35,36^ The findings of our study suggest that CHWs had a significant effect on preventing readmissions during or after short rehabilitation stays. Potential reasons for this effect may be that CHWs addressed unmet medical and social needs that occurred during the transition from rehabilitation to home and that CHWs improved communication among the patient, rehabilitation staff, and primary physician prior to return to home. While inpatients discharged to rehabilitation prior to transitioning home have been identified as high-risk for 30-day readmission, little research has been done to determine effective interventions to reduce readmissions. Results from our post hoc analyses demonstrated that CHWs are a promising intervention for this population, but future studies are needed to confirm these findings.

Most CHW-focused trials have not demonstrated significant reductions in hospital readmissions. However, a 2020 pooled analysis of 3 CHW randomized clinical trials^37^ found a combined significance in reduced hospital readmissions. The Community Care Transitions intervention used in this study differed from prior studies in that all participants were affiliated with a hospital ACO insurance benefit and had a PCP, a working phone number, and a residential address within a specific radius. Preexisting participant connections to primary care and the ACO network were essential for effective communication between CHWs and clinical teams and for assisting participants with access to postdischarge care. This was key to CHWs connecting participants with resources and programs when they needed them. As in most prior published trials, CHWs in our study received unique training focused on reaching patients after discharge and identifying resources for patient health-related social needs. Each of these elements likely contributed to improved clinical outcomes.

The results of this randomized clinical trial also emphasized that even in an ACO where there are more resources than in prior published CHW-intervention focused trials, readmission rates for control participants were relatively high. Although an equal proportion of intervention and control participants were enrolled in comprehensive case management or nursing programs as a part of usual care in an ACO, adding CHW care significantly reduced readmission rates. This difference highlights CHW capacity to address gaps in care related to unmet psychosocial needs (eg, securing meal delivery, transportation, access to medications, elder care services, accompaniment to clinic visits). CHWs can add value to the ACO model in a large health care organization by better connecting patients to community resources and programs as well as primary and subspecialty clinical homes.

Overall, there was a reduction of more than 30 percentage points in missed appointments among intervention participants 30 days after discharge compared with control participants. This effect was sustained in the subgroup analysis for participants discharged to rehabilitation and home. This difference has been demonstrated in prior care transitions studies^38,39^ and underlines the efficacy of CHWs in improving postdischarge follow-up. Baseline and postintervention period surveys demonstrated a reduction in perceived likelihood of hospital readmission for intervention participants, although this reduction was not statistically significantly different from the control group. This suggests that CHWs may promote increased competence, knowledge, and engagement influencing perceptions about readmission.^40,41^

The most frequent CHW activities were psychosocial support and reinforcement of adherence to care plans. These activities were similar to those described in studies by Kangovi et al^21^ and Wells et al,^23^ but most studies to date have not established a standard of care for CHW interventions, making direct comparisons between studies challenging. This represents an area of opportunity to more completely categorize and describe CHW outreach as part of an evidence base.^42,43,44,45^

### Limitations

This study has limitations. Despite use of validated self-reported measures of health care utilization in our 30-day postdischarge survey, we may not have identified all encounters occurring outside our hospital system. However, the fact that all participants were within the ACO network helped ensure that enrolled participants received most, if not all, of their care within designated ACO coverage. Also, healthy user bias, with patients who were the most ill being unable to enroll, may have resulted in underrepresentation of patients with even higher rates of medical complexity. While we considered individuals discharged to rehabilitation to be a legitimate group to study, study participants were enrolled and randomized while hospitalized, and it was not possible to determine who was going to rehabilitation vs home until the time of discharge. Therefore, we were unable to stratify on this characteristic at the time of randomization. Approximately 32% of 1280 patients identified as eligible for the study were unable to be enrolled owing to being busy with clinical care or discharged prior to meeting with study staff. In the future, this could be addressed by having CHWs contact patients via phone after discharge. We were unable to enroll non–English-speaking participants because of limited funding for bilingual study materials and staff. The study was conducted at a single urban hospital and enrolled ACO-insured inpatients with a working telephone and who lived within a 20-mile hospital radius. Therefore, study findings may not be generalizable to patients who have non-ACO health insurance, are uninsured, do not speak English, do not have a working telephone, or live in rural settings.

## Conclusions

This randomized clinical trial found that integration of CHWs into clinical care improved preventive care and reduced 30-day readmissions for patients discharged to rehabilitation. Reducing preventable readmissions is a central priority for the Centers for Medicare & Medicaid Services and principal health care stakeholders. As US health care organizations continue to adopt ACO models with the goal of achieving higher quality care at lower costs, policies supporting insurance-based reimbursement for CHW care and investment in comprehensive training and integration of CHWs as valued members of patient care teams will continue to be critical.^46^ Future research is needed to identify which patients benefit most from certain CHW intervention activities.

## Notes

Supplement 1. Trial Protocol

Supplement 2. **eAppendix.** Summary of IRB Protocol Changes **eTable 1.** Adjusted and Unadjusted Analysis for Clinical Outcomes of All Intervention and Control Participants **eTable 2.** Discharge Disposition Subgroup Analysis With Clinical Outcomes for Intervention and Control Participants Discharged to Rehabilitation vs Home **eTable 3.** Adjusted and Unadjusted Analysis for Clinical Outcomes of 127 Participants Discharged to Short-Stay Rehabilitation Prior to Home **eTable 4.** Adjusted and Unadjusted Analysis for Clinical Outcomes of 423 Participants Discharged Home

Supplement 3. Data Sharing Statement
