# Supplementary material for: Effect of Community Health Workers on 30-Day Hospital Readmissions in an Accountable Care Organization Population: A Randomized Clinical Trial
Source: JAMA Netw Open. 2021 May 20;4(5):e2110936. doi: 10.1001/jamanetworkopen.2021.10936 (PMC8138690; doi:10.1001/jamanetworkopen.2021.10936)
Supplement: Supplement 1. — Trial Protocol [file jamanetwopen-e2110936-s001.pdf]

PARTNERS HUMAN RESEARCH COMMITTEE
PROTOCOL SUMMARY

Answer all questions accurately and completely in order to provide the PHRC with the relevant information to assess the risk-benefit ratio for the study. <u>Do not leave sections blank.</u>

PRINCIPAL/OVERALL INVESTIGATOR

Jocelyn A. Carter, MD, MPH

PROTOCOL TITLE

Implementing 30-day Post- Discharge Patient-Community Health Worker Pairings

FUNDING

Department: General Internal Medicine 30KA-MGH

PeopleSoft Fund # 230199

InfoEd Proposal Number **2017A050810**

VERSION DATE

3.12.2017

SPECIFIC AIMS

Concisely state the objectives of the study and the hypothesis being tested.

This study is a randomized controlled trial of 1200 adult patients admitted to internal medicine units White 9 and White 10 that will examine the effectiveness of pairing patients that are high risk for readmission with community health workers for thirty days after hospital discharge on hospital readmission rates, emergency room visits, adherence to post-discharge medical appontments, and satisfaction with care. During this study, community health workers will partner with patients for 30 days after discharge from the hospital to address barriers to care during phone contacts, home visits, and while accompanying patients to medical appointments and other non-clinical supportive care.

<u>AIM 1)</u> Determine if patients randomly assigned to the intervention group have lower rates of hospital readmission than the control group at the end of the intervention.

Hypothesis 1.1 Study participants in the intervention group will have lower rates of post-discharge thirty day readmission than the study participants in the control group at the end of the intervention.

<u>AIM 2)</u> Determine if patients randomly assigned to the intervention group will have less emergency room visits than the control group at the end of the intervention

Hypothesis 2.1 Study participants in the intervention group will have less emergency room visits *than the study participants in the control group at the end of the intervention.*

<u>AIM 3)</u> Determine if patients randomly assigned to the intervention group have more adherence to post-discharge appointments that study group participants in the control group at the end of the intervention

Hypothesis: 3.1 *Study participants in the intervention group will have* higher rates of attendance at post-discharge medical appointments *than the study participants in the control group at the end of the intervention.*

<u>AIM 4)</u> Determine if patients randomly assigned to the intervention group have higher levels of patient psychosocial support and satisfaction with post-discharge care than the control group

Hypothesis 4.1: Study participants in the intervention group will have higher levels of patient psychosocial support and satisfaction with post-discharge care *than the study participants in the control group at the end of the intervention.*

<u>AIM 5)</u> Determine if Primary Care Physicians of patients randomly assigned to the intervention group have higher levels of satisfaction with post-discharge care than Primary Care Providers of patients in the control group

Hypothesis 5.1: Primary Care Physicians of patients randomly assigned to the intervention group have higher levels of satisfaction with post-discharge care than Primary Care Providers of patients in the control group

BACKGROUND AND SIGNIFICANCE

Provide a brief paragraph summarizing prior experience important for understanding the proposed study and procedures.

In an era of health care reform with increasing emphasis on higher quality healthcare at lower costs, hospital-associated processes of care and outcomes have been examined under a magnifying lens. In 2011, there were approximately 3.3 million adult 30-day all-cause hospital readmissions in the United States generating $41.3 billion in hospital costs.[i] Up to $8.26 billion (15-20%) of this is considered to be potentially preventable.[ii] While reasons for elevated numbers of readmissions are considered to be multi-factorial, numerous studies have demonstrated the relationships between hospital readmissions and factors steeped in social determinants of health (SDH). Lack of education[iii,iv] socioeconomic status,[v,vi,vii,viii,ix, x] and lack of social support[xi, xii] have all been cited as core contributors to readmission rates and poor care transitions. Additional studies focused on disease, racial ethnicity, language and socioeconomic status have also been identified as drivers of poor health outcomes and increased rates of readmission.[xiii,xiv, xv,xvi,xvii,xviii]
The MGH Department of Medicine (DOM) cares for over 10,000 inpatients per year presenting with diagnoses identified by CMS as most commonly associated with 30-day hospital readmissions including CHF, septicemia and pneumonia. According to preliminary data from an ongoing MGH DOM readmissions outcomes study generated from 2 DOM units from 2012-2016, less than 45% of surveyed MGH DOM readmitted within 30 days of hospitalization have reading skills expected for basic interpretation of prescription labels and an estimated 22% fall within 200% of the defined poverty level. With the goal of achieving the IOM Crossing the Quality Chasm goals of providing better health, better outcomes and better value during every encounter,[xix] current MGH DOM best practices include

collaborating with primary care providers (PCPs) at the time of and during discharge, performance of warm-handoffs to PCPs and post- acute care facilities accepting discharged patients, making PCP/relevant sub-specialty appointments at the time of discharge, hospitalist continuity visits to patients when readmissions occur, nurse calls to patients within the first 24-48 hours after discharge, home telemetry and weight monitoring, and utilization of VNA, home physical/occupational therapy, and elder services. Despite these practices, MGH 30- day readmission rates remain sub-optimal at 18% as of June 2016.

RESEARCH DESIGN AND METHODS

Briefly describe study design and anticipated enrollment, i.e., number of subjects to be enrolled by researchers study-wide and by Partners researchers. Provide a brief summary of the eligibility criteria (for example, age range, gender, medical condition). Include any local site restrictions, for example, "Enrollment at Partners will be limited to adults although the sponsor's protocol is open to both children and adults."

The experimental design is a randomized controlled trial. Eligibility amongst adult patients (~1200 adults age 18-109 years) admitted to the Massachusetts General Hospital (MGH) White 9 and White 10 inpatient medicine units who and meet all eligibility criteria will be considered. The following inclusion criteria will be applied. Patients must be 1) identified as high risk for readmission per the QPID readmission risk e-tool, 2) enrolled in a Partners Risk Contract, 3) living within 15 miles of MGH (55 Fruit Street, Boston, MA 02114), 4) have a working home/mobile telephone number where they can be reached 5) English speaking and 6) have the ability to consent to study participation. The following exclusion criteria will be applied. Exclusion will apply to patients that 1) homeless at the time of admission 2) do not have cognition to complete the survey or require caregiver prompting or response for questionnaire completion 3) have a history of known lack of capacity to consent (due to guardianship or invoked health care proxy). We plan to enroll 1200 study participants (600 in the intervention arm and 600 in the control arm). The estimated sample size is based on the minimum number of people required to demonstrate a statistically significant difference between those that receive the intervention and those that do not. It also represents the maximum number of participants that can be reliably seen by the community health workers supported by the Partners funding.

Briefly describe study procedures. Include any local site restrictions, for example, "Subjects enrolled at Partners will not participate in the pharmacokinetic portion of the study." Describe study endpoints.

After reviewing the fact sheet and consenting to enrollment by signing the informed consent form, patients will randomized to the intervention or control groups of the study.

Intervention Arm Group:
Patients randomized to the intervention group will be paired with community health workers prior to discharge from the hospital and patient- centered program goals will be established. A patient questionnaire (10-15 minutes) will be administered to intervention study participants by a study coordinator prior to discharge. A chart review will be performed for all intervention group participants by study coordinators prior to patient discharge. Patient-CHW pairings will continue for thirty days post-discharge and will include phone contacts, home visits, accompanying patients to medical appointments and other non-clinical supportive care. Both intervention group participants and primary care providers (PCPs) of intervention study participants will complete questionnaires 30-60 days after hospital

discharge to assess certain patient (10 minute questionnaire; via phone) and primary care provider (5 minute questionnaire via email) perceptions (Please see Aim 4 and Aim 5). CHWs will document patient encounters in a REDCap database and complete patient care notes in EPIC. All intervention study participants readmitted within thirty days of prior discharge will be administered a readmission questionnaire by study coordinators. A chart review for readmitted study participants will also be performed by study staff. A REDCap database will be used to store all questionnaire and chart review data. Readmission rates, ED visits, PCP appointment compliance, patient/ PCP will be tracked.

Control Arm Group:
Those randomized to the control group will receive usual care without a community heath worker pairing. A patient questionnaire (10-15 minutes) will be administered to intervention study participants by a study coordinator prior to discharge. A chart review will be performed for all control group participants by study coordinators prior to patient discharge. Both control group participants and primary care providers (PCPs) of intervention study participants will complete questionnaires 30-60 days after hospital discharge to assess certain patient (10 minute questionnaire; via phone) and primary care provider (5 minute questionnaire via email) perceptions (Please see Aim 4 and Aim 5). A REDCap database will be used to store all questionnaire and chart review data. Readmission rates, ED visits, PCP appointment compliance, patient/ PCP perceptions will be tracked.

Three groups of research subjects will be assessed: the intervention study group, the control study group, and the PCPs of the patients enrolled in the study.

For studies involving treatment or diagnosis, provide information about standard of care at Partners (e.g., BWH, MGH) and indicate how the study procedures differ from standard care. Provide information on available alternative treatments, procedures, or methods of diagnosis.

This study does not involve treatment or diagnostic processes.

Describe how risks to subjects are minimized, for example, by using procedures which are consistent with sound research design and which do not unnecessarily expose subjects to risk or by using procedures already being performed on the subject for diagnostic or treatment purposes.

Minimal risk is anticipated in this study since no drugs or biologics are involved. All adverse events in association with the study will be reported to the Partners Institutional Review Board and according to Partners Healthcare/MGH policy.

Describe explicitly the methods for ensuring the safety of subjects. Provide objective criteria for removing a subject from the study, for example, objective criteria for worsening disease/lack of improvement and/or unacceptable adverse events. The inclusion of objective drop criteria is especially important in studies designed with placebo control groups.

While minimal safety risk is expected during this study, this study is not designed to detract from or interfere with clinical care. Individuals unable or unwilling to participate in survey or intervention processes will be withdrawn from the study.

FORESEEABLE RISKS AND DISCOMFORTS

Provide a brief description of any foreseeable risks and discomforts to subjects. Include those related to drugs/devices/procedures being studied and/or administered/performed solely for research purposes. In addition, include psychosocial risks, and risks related to privacy and confidentiality. When applicable, describe risks to a developing fetus or nursing infant.

No drugs, devices or biologic procedures will be administered. There are no forseeable discomforts or psychosocial risks to subjects. If after consenting to the study, study participants are no longer interested in being included in the study or are unable to complete the study for any reason, they will be withdrawn from the study. If there are any questions that make study participants uncomfortable, they may skip these questions. To minimize the risk to privacy and confidentiality, all study participants will be assigned a unique study identification number as part of a Partners password protected REDCap database. Only de-identified data will be analyzed and published. Data will only be assessable to authorized study staff including the PI, co-investigators and statistical staff.

EXPECTED BENEFITS

Describe both the expected benefits to individual subjects participating in the research and the importance of the knowledge that may reasonably be expected to result from the study. Provide a brief, realistic summary of potential benefits to subjects, for example, "It is hoped that the treatment will result in a partial reduction in tumor size in at least 25% of the enrolled subjects." Indicate how the results of the study will benefit future patients with the disease/condition being studied and/or society, e.g., through increased knowledge of human physiology or behavior, improved safety, or technological advances.

Study subjects may not receive direct benefit from study enrollment. However, it is possible that patients in the intervention group will have reduced rates thirty day readmissions, reduced ED visits, increased patient compliance with post-discharge appointments, higher levels of patient perceived psychosocial support and satisfaction with care. If this study demonstrates the expected benefit, this type of intervention may be spread to other parts of the hospital and beyond.

EQUITABLE SELECTION OF SUBJECTS

The risks and benefits of the research must be fairly distributed among the populations that stand to benefit from it. No group of persons, for example, men, women, pregnant women, children, and minorities, should be categorically excluded from the research without a good scientific or ethical reason to do so. Please provide the basis for concluding that the study population is representative of the population that stands to potentially benefit from this research.

Those that are unable to consent to the study and those that have prisoner status (with implicit loss of ability to consent independently) will be systematically excluded from the study. The remaining patients admitted to the pre-designated units of implementation during the study period will be considered for eligibility and identified for potential enrollment based on readmission risk e-tool scoring and the inclusion and exclusion criteria described above. As such, the study population is representative of the available population at risk for readmission and stands to benefit from this research.

When people who do not speak English are excluded from participation in the research, provide the scientific rationale for doing so. Individuals who do not speak English should not be denied

participation in research simply because it is inconvenient to translate the consent form in different languages and to have an interpreter present.

Active efforts are being made to create questionnaires in another language (Spanish). If questionnaires in this language are not available at the time of the start of the study, interpreter services will be utilized to obtain consent and conduct patient interviews to obtain the questionnaire data.

For guidance, refer to the following Partners policy:

Obtaining and Documenting Informed Consent of Subjects who do not Speak English
http://healthcare.partners.org/phsirb/nonengco.htm

RECRUITMENT PROCEDURES

Explain in detail the specific methodology that will be used to recruit subjects. Specifically address how, when, where and by whom subjects will be identified and approached about participation. Include any specific recruitment methods used to enhance recruitment of women and minorities.

Patients considered for enrollment will be pre-identified during multidisciplinary rounds that occur daily on each unit. During multi-disciplinary rounds, the case managers, attending nurses, bedside nurses and physician leads will review the barriers to discharge along with risk for readmission calculated by the QPID readmission risk e-tool. For the purposes of this study, community health workers will attend multidisciplinary rounds and for those patients that meet study eligibility criteria, permission to approach patients will be obtained from the care team/bedside nurse. After permission is obtained, eligible patients will be approached by study coordinators who will describe the study to patients. If patients are willing to speak to a research team member about the study, the nature of the study and expected involvement will be described. A study fact sheet as well as the informed consent letter detailing the protocol will be distributed to patients at that time. Patients will have an opportunity to read all materials, ask questions and engage in discussion about the study details. All patient consent processes will emphasize that the medical care is not contingent on participation in the study. If the patient is not interested and/or consent is not obtained for participation, the patient will not be enrolled. If, after hearing about the study and receiving the study fact sheet, a patient is interested in participating in the study, the patient may sign the consent form and be enrolled in the study. Patients that are undecided on study participation may take additional time to consider enrollment up until the time of hospital discharge. Consenting patients that are randomized to the intervention arm will be paired with a CHW who will meet with them prior to hospital discharge.

No other modes of recruitment will be utilized. No specific recruitment methods will be used to enhance the recruitment of women or minorities.

Provide details of remuneration, when applicable. Even when subjects may derive medical benefit from participation, it is often the case that extra hospital visits, meals at the hospital, parking fees or other inconveniences will result in additional out-of-pocket expenses related to study participation. Investigators may wish to consider providing reimbursement for such expenses when funding is available

No remuneration will be involved in this study.

For guidance, refer to the following Partners policies:

Recruitment of Research Subjects
http://healthcare.partners.org/phsirb/recruit.htm

Guidelines for Advertisements for Recruiting Subjects
http://healthcare.partners.org/phsirb/advert.htm

Remuneration for Research Subjects
http://healthcare.partners.org/phsirb/remun.htm

CONSENT PROCEDURES

Explain in detail how, when, where, and by whom consent is obtained, and the timing of consent (i.e., how long subjects will be given to consider participation). For most studies involving more than minimal risk and all studies involving investigational drugs/devices, a licensed physician investigator must obtain informed consent. When subjects are to be enrolled from among the investigators' own patients, describe how the potential for coercion will be avoided.

After permission is obtained, eligible patients will be approached by study coordinators who will describe the study to patients. If willing to speak to a research team member about the study, the nature of the study and expected involvement will be described. A study fact sheet as well as the informed consent letter detailing the protocol will be distributed to patients at that time. Patients will have an opportunity to read all materials, ask questions and engage in discussion about the study details. During the informed consent process, it will be explained that consenting study participants will be randomized to the intervention group or the control group with a 50% chance of being assigned to the intervention. Patients will also be told that if assigned to the intervention study group, they will meet and be paired with a CHW for thirty days after discharge, and receive supportive care from CHWs (including calls, home visits, and accompaniment to medical appointments). Patients will be told that if assigned to the intervention group they will complete a pre-discharge questionnaire prior to hospital discharge, complete a post-discharge questionnaire thirty days after discharge and undergo a chart review while hospitalized. Patients will also be told that if assigned to the intervention study group and readmitted within thirty days of hospital discharge, they will take a readmission questionnaire prior to leaving the hospital and undergo a chart review while hospitalized for readmission. Patients will be informed that if they are assigned to the control group they will complete a pre-discharge questionnaire prior to hospital discharge, complete a post-discharge questionnaire thirty days after discharge and undergo a chart review while hospitalized. Patients will also be told that if assigned to the control study group and readmitted within thirty days of hospital discharge, they will take a readmission questionnaire prior to leaving the hospital and undergo a chart review while hospitalized.

The fact sheet will distill the purpose and main components of the study in a simplified way so that patients can get an idea of what the study will involve by reading a single page. The consent form will describe all questionnaire and chart review procedures as well as the timing of dissemination to patients. Patients that are undecided on study participation may take additional time to consider enrollment up until the time of hospital discharge. Patients interested in participating in the study after reviewing the fact sheet and consenting to enrollment by signing the informed consent form, will be enrolled in the study.

NOTE: When subjects are unable to give consent due to age (minors) or impaired decision-making capacity, complete the forms for Research Involving Children as Subjects of Research and/or Research Involving Individuals with Impaired Decision-making Capacity, available on the New Submissions page on the PHRC website:
http://healthcare.partners.org/phsirb/newapp.htm#Newapp

For guidance, refer to the following Partners policy:
Informed Consent of Research Subjects
http://healthcare.partners.org/phsirb/infcons.htm

DATA AND SAFETY MONITORING

Describe the plan for monitoring the data to ensure the safety of subjects. The plan should include a brief description of (1) the safety and/or efficacy data that will be reviewed; (2) the planned frequency of review; and (3) who will be responsible for this review and for determining whether the research should be altered or stopped. Include a brief description of any stopping rules for the study, when appropriate. Depending upon the risk, size and complexity of the study, the investigator, an expert group, an independent Data and Safety Monitoring Board (DSMB) or others might be assigned primary responsibility for this monitoring activity.

NOTE: Regardless of data and safety monitoring plans by the sponsor or others, the principal investigator is ultimately responsible for protecting the rights, safety, and welfare of subjects under his/her care.

While the anticipated risks requiring study discontinuation are quite low, the study may be stopped at any time if a safety or efficacy compromise occurs. The primary responsibility for monitoring data and patient safety will be that of the PI, Dr. Carter, each month. There is no expectation for need of a Data and Safety Monitoring Board. All complaints about the study will be reviewed by Dr. Carter and discussed with the study co-investigators.

Describe the plan to be followed by the Principal Investigator/study staff for review of adverse events experienced by subjects under his/her care, and when applicable, for review of sponsor safety reports and DSMB reports. Describe the plan for reporting adverse events to the sponsor and the Partners' IRB and, when applicable, for submitting sponsor safety reports and DSMB reports to the Partners' IRBs. When the investigator is also the sponsor of the IND/IDE, include the plan for reporting of adverse events to the FDA and, when applicable, to investigators at other sites.

NOTE: In addition to the adverse event reporting requirements of the sponsor, the principal investigator must follow the Partners Human Research Committee guidelines for Adverse Event Reporting

While adverse events are not anticipated a review of data will occur monthly by Dr. Carter and all adverse events will be discussed with the IRB. Dr. Carter, the PI, will be responsible for maintaining the integrity of the study database.

MONITORING AND QUALITY ASSURANCE

Describe the plan to be followed by the principal investigator/study staff to monitor and assure the validity and integrity of the data and adherence to the IRB-approved protocol. Specify who will be responsible for monitoring, and the planned frequency of monitoring. For example, specify who will review the accuracy and completeness of case report form entries, source documents, and informed consent.

NOTE: Regardless of monitoring plans by the sponsor or others, the principal investigator is ultimately responsible for ensuring that the study is conducted at his/her investigative site in accordance with the IRB-approved protocol, and applicable regulations and requirements of the IRB.

Data review and database review will be completed by the PI, Dr. Carter, each month to ensure the validity and integrity of the data generated in accordance with all study procedures described in the IRB-approved protocol.

For guidance, refer to the following Partners policies:

Data and Safety Monitoring Plans and Quality Assurance
http://healthcare.partners.org/phsirb/datasafe.htm

Adverse Event Reporting Guidelines
http://healthcare.partners.org/phsirb/adverse_events.htm

PRIVACY AND CONFIDENTIALITY

Describe methods used to protect the privacy of subjects and maintain confidentiality of data collected. This typically includes such practices as substituting codes for names and/or medical record numbers; removing face sheets or other identifiers from completed surveys/questionnaires; proper disposal of printed computer data; limited access to study data; use of password-protected computer databases; training for research staff on the importance of confidentiality of data, and storing research records in a secure location.

NOTE: Additional measures, such as obtaining a Certificate of Confidentiality, should be considered and are strongly encouraged when the research involves the collection of sensitive data, such as sexual, criminal or illegal behaviors.

The electronic database generated will be password-protected and accessed only by authorized study staff trained on the importance data confidentiality and record security (limited to the PI, co-investigators and statistical lead). There are no foreseen risks to privacy and all study participants will be assigned a unique study identification number as part of a Partners password protected REDCap database. Only de-identified data will be analyzed and published.

SENDING SPECIMENS/DATA TO RESEARCH COLLABORATORS OUTSIDE PARTNERS

Specimens or data collected by Partners investigators will be sent to research collaborators outside Partners, indicate to whom specimens/data will be sent, what information will be sent, and whether the specimens/data will contain identifiers that could be used by the outside collaborators to link the specimens/data to individual subjects.

No specimens/data will be collected or sent in this study

Specifically address whether specimens/data will be stored at collaborating sites outside Partners for future use not described in the protocol. Include whether subjects can withdraw their specimens/data, and how they would do so. When appropriate, submit documentation of IRB approval from the recipient institution.

No specimens/data will be collected or stored in this study

RECEIVING SPECIMENS/DATA FROM RESEARCH COLLABORATORS OUTSIDE PARTNERS

When specimens or data collected by research collaborators outside Partners will be sent to Partners investigators, indicate from where the specimens/data will be obtained and whether the specimens/data will contain identifiers that could be used by Partners investigators to link the specimens/data to individual subjects. When appropriate, submit documentation of IRB approval and a copy of the IRB-approved consent form from the institution where the specimens/data were collected.

No specimens/data will be shared with research collaborators outside partners

---

[i] Hines AL (Truven Health Analytics), Barrett ML (ML Barrett, Inc), Jiang HJ (AHRQ), and Steiner CA (AHRQ). Conditions With the Largest Number of Adult Hospital Readmissions by Payer, 2011. HCUP Statistical Brief #172. April 2014. Agency for Healthcare Research and Quality, Rockville, MD. http://www.hcup-us.ahrq.gov/reports/statbriefs/sb172-Conditions-Readmissions-Payer.pdf.

[ii] Goldfield NI, McCullough E, Hughes JS, Tang AM, Eastman B, Rawlins LK, and Averill RF. (2008) Identifying Potentially Preventable Readmissions. Heath Care Review, 30(1), 75-91

[iii] Arbaje, A. I., Wolff, J. L., Yu, Q. L., Powe, N. R., Anderson, G. F., & Boult, C. (2008). Postdischarge environmental and socioeconomic factors and the likelihood of early hospital readmission among community-dwelling Medicare beneficiaries. Gerontologist, 48(4), 495–504.

[iv] Calvillo-King, L., Arnold, D., Eubank, K.J., Lo, M., Yunyongying, P., Stieglitz, H., & Halm, E. A. (2013) Impact of social factors on risk of readmission or mortality in pneumonia and heart failure: systematic review. Journal of General Internal Medicine, 28(2), 269-82.

[v] Bernheim, S. M., Spertus, J. A., Reid, K. J., Bradley, E. H., Desai, R. A., Peterson, E. D., Rathore, S. S., Norman, S.-L. T., Jones, P. G., Rahimi, A., & Krumholz, H. M. (2007). Socioeconomic disparities in outcomes after acute myocardial infarction. American Heart Journal, 153(2), 313–319.

[vi] Tsuchiahashi, M., Tsutsui, H., Kodama K, Kasagi, F., Setoguchi, S., Mohr, M., Kubota, T., Takeshita, A. (2001). Medical and socioenvironmental predictors of hospital readmission in patients with congestive heart failure. American Heart Journal.142 (4), E7.

[vii] Philbin, E.F., Dec, G.W., Jenkins, P.L. & DiSalvo, T.G. (2001). Socioeconomic status as an independent risk factor for hospital readmission for heart failure. American Journal of Cardiology, 87(12),1367–71.

[viii] Billings J, Zeitel L, Lukomnik J, Carey, T.S., Blank, A.E., Newman, L. (1993). Impact of socioeconomic status on hospital use in New York City. Health Aff,12 (1), 162–173.

[ix] Rathore, S.S., Masoudi, A.F., Wang, Y., Curtis, P.J., Foody, M.J., Havranek, P.E., & Krumholz, M.H. (2006). Socioeconomic staturs, treatment, and outcomes among elderly patients hospitalized with heart failure: Findings from the National Heart Failure Project. Am. Heart J., 152(2), 371-378.

[x] Joynt, E.K., & Jha, K.A. (2013). A path forward on Medicare readmissions. New England Journal of Medicine, 368(1), 1175-1177.

[xi] Vinson, M.J., Rich, W.M., Sperry, C.J., Shah, S.A., & McNamara, T. (1990). Early readmission of elderly patients with congestive heart failure. Journal of the American Geriatrics Society, 38(12), 1290-1295.

[xii] Hu, J., Gonsahn, M.D., & Nerenz, D.R. (2014). Socioeconomic status and readmission: Evidence from an urban teaching hospital. Health Aff (Millwood)., 33(5):778-785.

[xiii] Jencks S.F., Williams M.V., & Coleman E.A. (2009). Rehospitalizations among patients in the Medicare fee-for-service program. New England Journal of Medicine, 360(14), 1418-1428.

[xiv] Allaudeen N., Vidyarthi A., Maselli J., & Auerbach A. (2011). Redefining readmission risk factors for general medicine patients. J Hosp Med., 6(2), 54-60.

[xv] Joynt K.E., Orav E.J., & Jha A.K. (2011). Thirty-day readmission rates for Medicare beneficiaries by race and site of care. JAMA, 305(7), 675-681.

[xvi] Aranda J.M., Johnson J.W., & Conti J.B. (2009). Current trends in heart failure readmission rates: Analysis of Medicare data. Clin Cardiol. 32(1), 47-52.

[xvii] Karliner L.S., Kim S.E., Meltzer D.O., & Auerbach A.D. (2010). Influence of language barriers on outcomes of hospital care for general medicine inpatients. J Hosp Med., 5(5), 276-282.

[xviii] Osei-Anto A., Joshi M., Audet A., Berman A., & Jencks S. (2010) Health Care Leader Action Guide to Reduce Avoidable Readmissions. Chicago, IL: Health Research & Educational Trust.

[xix] Institute of Medicine (IOM). (2001). Crossing the Quality Chasm: A New Health System for the 21st Century. Washington, D.C: National Academy Press.
