# Supplementary material for: Effect of Community Health Workers on 30-Day Hospital Readmissions in an Accountable Care Organization Population: A Randomized Clinical Trial
Source: JAMA Netw Open. 2021 May 20;4(5):e2110936. doi: 10.1001/jamanetworkopen.2021.10936 (PMC8138690; doi:10.1001/jamanetworkopen.2021.10936)
Supplement: Supplement 2. — eAppendix. Summary of IRB Protocol Changes eTable 1. Adjusted and Unadjusted Analysis for Clinical Outcomes of All Intervention and Control Participants eTable 2. Discharge Disposition Subgroup Analysis With Clinical Outcomes for Intervention and Control Participants Discharged to Rehabilitation vs Home eTable 3. Adjusted and Unadjusted Analysis for Clinical Outcomes of 127 Participants Discharged to Short-Stay Rehabilitation Prior to Home eTable 4. Adjusted and Unadjusted Analysis for Clinical Outcomes of 423 Participants Discharged Home [file jamanetwopen-e2110936-s002.pdf]

Supplemental Online Content

Carter J, Hassan S, Walton A, Yu L, Donelan K, Thorndike AN. Effect of community health workers on 30-day hospital readmissions in an accountable care organization population: a randomized clinical trial. *JAMA Netw Open*. 2021;4(5):e2110936. doi:10.1001/jamanetworkopen.2021.10936

eAppendix. Summary of IRB Protocol Changes

eTable 1. Adjusted and Unadjusted Analysis for Clinical Outcomes of All Intervention and Control Participants

eTable 2. Discharge Disposition Subgroup Analysis With Clinical Outcomes for Intervention and Control Participants Discharged to Rehabilitation vs Home

eTable 3. Adjusted and Unadjusted Analysis for Clinical Outcomes of 127 Participants Discharged to Short-Stay Rehabilitation Prior to Home

eTable 4. Adjusted and Unadjusted Analysis for Clinical Outcomes of 423 Participants Discharged Home

This supplemental material has been provided by the authors to give readers additional information about their work.

© 2021 Carter J et al. *JAMA Network Open.*

eAppendix. Summary of IRB Protocol Changes

Date of Proposed Change	Date Accepted by IRB	Description of Change
March 6, 2017	April 6, 2017	Staff amendment
April 18, 2017	April 26, 2017	Staff amendment
April 28, 2017	May 12, 2017	More explicit description of the hospital database identifying patients as high risk for readmission with ≥2 hospitalizations in the prior 6 months or >3 hospitalizations the prior 12 months; add a more explicit description of Partners Healthcare patients to eligibility criteria
May 20, 2017	June 2, 2017	Staff amendment
June 1, 2017	June 12 2017	Staff amendment
June 8, 2017	June 29, 2017	Staff amendment
June 8, 2017	June 29, 2017	Staff amendment
August 19, 2017	August 30, 2017	Extension of study to additional floors to meet target enrollment
August 19, 2017	September 4, 2017	Staff amendment
September 11, 2017	September 13, 2017	Staff amendment
January 23, 2018	January 26, 2018	Staff amendment
January 14, 2018	February 5 2018	Staff amendment
October 29, 2018	November 2, 2018	Staff amendment
August 6, 2018	January 17, 2019	Staff amendment
January 31, 2019	January 31, 2019	Staff amendment
June 11, 2019	June 26, 2019	Staff amendment
October 13, 2019	October 13, 2019	Staff amendment
January 19, 2020	January 27, 2020	Staff amendment
June 9, 2020	June 9, 2020	Staff amendment

© 2021 Carter J et al. *JAMA Network Open.*

eTable 1. Adjusted and Unadjusted Analysis for Clinical Outcomes of All Intervention and Control Participants

	Control (n-273) N (%)	Intervention (n-277) N (%)	P value	Unadjusted Odds Ratio (95% CI)	Adjusted Odds Ratio * (95% CI)
Hospital Readmissions	67 (24.5)	35 (12.6)	0.0003	0.44 (0.28, 0.70)	0.45 (0.29, 0.72)
Missed Primary Care or Specialty Appts	92 (33.7)	61 (22.0)	0.002	0.56 (0.38, 0.81)	0.56 (0.38, 0.82)
Emergency Department Visits	46 (16.8)	31 (11.2)	0.056	0.62 (0.38, 1.02)	0.62 (0.38, 1.02)

*Covariates include age, race, sex, number of hospitalizations, insurance, living alone, and discharge disposition

eTable 2. Discharge Disposition Subgroup Analysis With Clinical Outcomes for Intervention and Control Participants Discharged to Rehabilitation vs Home

	Home			Short-term Rehabilitation		
	Control	Intervention	P	Control	Intervention	P
N	206	217		67	60	
Readmission %	20.4	14.7	0.13	37.3	5.0	<0.0001
ED visit %	15.5	12.0	0.29	20.9	8.3	0.048
Miss appointment %	33.0	22.1	0.012	35.8	21.7	0.08

© 2021 Carter J et al. *JAMA Network Open.*

eTable 3. Adjusted and Unadjusted Analysis for Clinical Outcomes of 127 Participants Discharged to Short-Stay Rehabilitation Prior to Home

	Control (n=67) N (%)	Intervention (n=60) N (%)	P value	Unadjusted Odds Ratio (95% CI)	Adjusted Odds Ratio * (95% CI)
Hospital Readmissions	25 (37.3)	3 (5)	0.0002	0.09 (0.03, 0.31)	0.08 (0.02, 0.30)
Missed Primary Care or Specialty Appts	24 (35.8)	13 (21.7)	0.048	0.50 (0.22, 1.09)	0.39 (0.16, 0.96)
Emergency Department Visits	14 (20.9)	5 (8.3)	0.08	0.34 (0.12, 1.02)	0.33 (0.11, 1.06)

*Covariates include age, race, sex, number of hospitalizations, insurance, living alone

eTable 4. Adjusted and Unadjusted Analysis for Clinical Outcomes of 423 Participants Discharged Home

	Control (n=206) N (%)	Intervention (n=217) N (%)	P value	Unadjusted Odds Ratio (95% CI)	Adjusted Odds Ratio* (95% CI)
Hospital Readmissions	42 (20.4)	32 (14.7)	0.13	0.68 (0.41, 1.12)	0.68 (0.41, 1.15)
Missed Primary Care or Specialty Appts	68 (33.0)	48 (22.1)	0.012	0.58 (0.37, 0.89)	0.57 (0.37, 0.89)
Emergency Department Visits	32 (15.5)	26(12.0)	0.29	0.74 (0.42, 1.29)	0.76 (0.43, 1.33)

*Covariates include age, race, sex, number of hospitalizations, insurance, living alone

© 2021 Carter J et al. *JAMA Network Open.*
