# Supplementary material for: Effect of Community Health Workers on 30-Day Hospital Readmissions in an Accountable Care Organization Population: A Randomized Clinical Trial
Source: JAMA Netw Open. 2021 May 20;4(5):e2110936. doi: 10.1001/jamanetworkopen.2021.10936 (PMC8138690; doi:10.1001/jamanetworkopen.2021.10936)
Supplement: Supplement 3. — Data Sharing Statement [file jamanetwopen-e2110936-s003.pdf]

Data Sharing Statement

Carter. Effect of Community Health Workers on 30-Day Hospital Readmissions in an Accountable Care Organization Population. *JAMA Netw Open*. Published May 20, 2021. doi:10.1001/jamanetworkopen.2021.10936

Data
Data available: Yes
Data types: Deidentified participant data, Data dictionary
How to access data: Upon request to corresponding author Jocelyn Carter (jcarter0@mgh.harvard.edu)
When available: With publication

Supporting Documents
Document types: None

Additional Information
Who can access the data: Researchers whose proposed use of the data has been approved
Types of analyses: For specified purpose presented at the time of the request
Mechanisms of data availability: After approval of a proposal and with a signed data access agreement
